# Outcomes of inguinal hernia repair in octogenarians: A propensity score–matched analysis of the Herniamed Registry

**DOI:** 10.1007/s10029-026-03689-5

**Published:** 2026-05-12

**Authors:** Ralph Lorenz, J. Conze, R. Fortelny, F. Köckerling, F. Mayer, H. Niebuhr, W. Reinpold, B. Stechemesser, R. Wiessner, D. Adolf, Ch. Paasch

**Affiliations:** 1 Hernia Center 3+CHIRURGEN, Klosterstrasse 34/35, 13581 Berlin, Germany; 2 Department of General and Abdominal Surgery, Clinic for General and Abdominal Surgery, University Hospital Brandenburg an der Havel, Hochstrasse 29, 14770 Brandenburg an der Havel, Germany; 3 UM Hernienzentrum Dr. Conze, Arabellastraße 17, 81925 München, Germany; 4 Department of General, Visceral, Pediatric and Transplant Surgery, University Hospital Aachen, Aachen, Germany; 5 Department of General Surgery, Klinik Ottakring, Montleartstraße 37, Vienna, A- 1160 Austria; 6 Medical Faculty, Sigmund Freud University, Freudplatz 3, Vienna, A-1020 Austria; 7 Hernia Center, Vivantes Humboldt Hospital, Academic Teaching Hospital of Charité, University Medicine, Am Nordgraben 2, 13509 Berlin, Germany; 8 Department of Surgery, University Hospital, Paracelsus Medical University, Müllner Hauptstrasse 48, Salzburg, 5020 Austria; 9 Hamburger Hernien Centrum, Eppendorfer Baum 8, 20249 Hamburg, Germany; 10 Hamburger Hernien Centrum, Harburg (an der Helios Mariahilf Klinik), Stader Str. 203c, 21075 Hamburg, Germany; 11 Hernia Center Cologne, PAN-Klinik, Zeppelinstrasse 1, 50667 Cologne, Germany; 12 Department of General Surgery, Kardinal Schwarzenberg Klinikum, Schwarzach im Pongau, Austria; 13 StatConsult GmbH, Am Fuchsberg 11, Magdeburg, Germany

**Keywords:** Inguinal hernia, Groin hernias, Octagenarians, Geriatric patients, Hernia characteristics

## Abstract

**Background:**

With increasing life expectancy, a growing proportion of inguinal hernia repairs is performed in elderly patients. Evidence regarding perioperative safety and long-term outcomes in octogenarians remains limited. This study compared perioperative outcomes and one-year follow-up between patients aged < 80 and ≥ 80 years using data from the Herniamed Registry.

**Methods:**

A total of 394,309 fully documented unilateral primary inguinal hernia repairs with valid one-year follow-up were extracted from the Herniamed Registry (data export October 2025). After applying predefined inclusion and plausibility criteria, 45,576 patients aged ≥ 80 years and 348,733 patients aged < 80 years were eligible for analysis. A 1:1 propensity score–matched analysis was performed using demographic, hernia-related, surgical, and comorbidity variables, with exact matching for sex, surgical approach, and procedure type. Outcomes included intraoperative, general, and postoperative complications, complication-related reoperations, and one-year endpoints (pain at rest, pain on exertion, pain requiring treatment, recurrence, trocar hernia, secondary hemorrhage, seroma, and infection). McNemar’s test and odds ratios were used for statistical comparison.

**Results:**

Before matching, octogenarians more frequently underwent emergency surgery (6.8% vs. 2.1%), presented with scrotal or femoral hernias, and were more often treated under local or spinal anesthesia. Propensity score matching yielded 44,550 matched pairs. After matching, patients aged ≥ 80 years showed significantly higher rates of general and postoperative complications, reoperations, secondary hemorrhage, and infection. Conversely, one-year pain outcomes and recurrence rates were significantly lower in octogenarians. No significant differences were observed for intraoperative complications, trocar hernia, or seroma.

**Conclusions:**

Octogenarians undergoing inguinal hernia repair exhibit increased perioperative risk but favorable one-year outcomes regarding pain and recurrence. These findings support the overall safety and effectiveness of inguinal hernia repair in advanced age, provided that perioperative risk is carefully managed. Elective surgery should not be withheld solely due to age, and early intervention may reduce the risk of emergency presentations and improve postoperative outcomes.

**Supplementary information:**

The online version contains supplementary material available at 10.1007/s10029-026-03689-5.

## Introduction

Inguinal hernia repair is one of the most commonly performed procedures in general surgery worldwide [1]. With increasing life expectancy and demographic changes, the proportion of elderly patients undergoing inguinal hernia repair continues to rise [2]. By 2050, the global population aged ≥ 80 years is projected to exceed 400 million, underscoring the increasing clinical importance of optimizing surgical strategies for this expanding patient population and of avoiding delayed intervention that may result in treatment under less favorable conditions.

Advanced age is frequently accompanied by increased surgical complexity. Elderly patients often present with multiple comorbidities, altered physiological reserves, and functional limitations that may influence perioperative risk and postoperative recovery [3–5]. Nevertheless, several studies have demonstrated that elective inguinal hernia repair can be performed safely in elderly patients, including octogenarians and nonagenarians, particularly when performed under local anaesthesia [6–8]. Local anaesthesia has been associated with lower complication rates, shorter recovery times, and improved cost-effectiveness, especially in frail patients [9, 10].

In contrast, emergency hernia surgery in elderly patients is consistently associated with substantially higher morbidity and mortality. Delayed treatment may result in incarceration or strangulation requiring bowel resection and prolonged hospitalization. Mortality rates of up to 13% have been reported in nonagenarians undergoing emergency hernia repair [11–13]. These findings emphasize the potential benefits of timely elective intervention.

The optimal surgical approach in elderly patients remains a subject of ongoing discussion. Laparoscopic techniques have been associated with reduced postoperative pain, faster recovery, and lower rates of chronic postoperative inguinal pain, without an increase in major complications in elderly populations [14–16]. However, some studies have reported higher rates of postoperative urinary retention and longer operative times with laparoscopic repair in selected elderly cohorts, underscoring the importance of individualized perioperative decision-making [17, 18].

Postoperative quality-of-life outcomes are generally favourable across age groups. Improvements in pain and physical functioning have been reported even in elderly patients following inguinal hernia repair [19, 20]. Interestingly, younger patients appear to have a higher risk of chronic postoperative inguinal pain, whereas older patients often report greater satisfaction and faster subjective recovery [7, 21].

Despite these findings, real-world data reflecting routine clinical practice in octogenarians remain limited. Large registry-based analyses may provide valuable insights into outcomes in this rapidly growing patient population. The present study therefore analyses data from the Herniamed Registry to evaluate perioperative and one-year outcomes of inguinal hernia repair in patients aged ≥ 80 years compared with younger patients. Specifically, we aimed to determine whether age ≥ 80 years is independently associated with perioperative complications and long-term outcomes after adjustment for patient characteristics, hernia features, and comorbidities.

## Materials and methods

### Herniamed registry

The Herniamed Registry is a multicentre, internet-based hernia registry established in 2009 with the aim of documenting hernia surgery and facilitating outcomes research [22]. Currently, 1053 hospitals and surgical practices in Germany, Austria, and Switzerland contribute data to the registry.

All patients provided written informed consent for participation. Patients were informed that postoperative complications should be reported to the treating hospital or surgical practice, which would conduct a clinical follow-up if necessary. Follow-up questionnaires are sent to patients and their general practitioners by the treating surgeon or hospital after 1, 5, and 10 years. These questionnaires collect information on postoperative complications and specifically assess pain at rest, pain during physical activity, and chronic pain requiring treatment. In addition, patients and their general practitioners are asked about the occurrence of recurrent inguinal hernias.

The Herniamed Registry has received ethical approval from the Ethics Committee of the Canton of St. Gallen (BASEC No. 2016 − 00123), the Ethics Committee of the University of Tübingen (287/2017 BO2), the Ethics Committee of the State Medical Association of Baden-Württemberg (F-2022-111), and the Ethics Committee of the University of Cologne (24-1115-retro).

### Study population and inclusion criteria

From the processed dataset comprising 1,542,456 cases, patients were selected according to the following predefined inclusion criteria:

- Inguinal hernia repair.
- Complete documentation of patient master data and operative data (mandatory fields completed).
- Age ≥ 18 years with valid age information.
- Unilateral procedures only.
- Primary hernia repairs only.
- Elective procedures (without incarceration) or emergency surgery.
- No use of a Physiomesh mesh.
- Laparoscopic procedures with mesh or open procedures.
- Date of surgery up to and including 31 August 2024, or later if one-year follow-up data were already available.
- Availability of a fully documented one-year follow-up.

After application of these criteria and plausibility checks, 394,309 patients were included in the analysis. Of these, 45,576 patients were aged ≥ 80 years (Table 1).

**Table 1:** Frequency distribution of age groups

Age ≥ 80 years	*N*	%
yes	45,576	11.6
no	348,733	88.4
Total	394,309	100

### Statistical analysis

All statistical analyses were performed using SAS version 9.4 (SAS Institute Inc., Cary, NC, USA) and intentionally calculated to a full significance level of 5% from an exploratory perspective. This means that and no adjustment for multiple testing was performed.

After verification of plausibility and inclusion criteria, pairwise comparisons were conducted between patients aged < 80 years and ≥ 80 years. Univariate analyses of the patient population were first performed with respect to the matching parameters. For unadjusted comparisons between age groups, the chi-square test and the robust t-test was used for categorical and continuous variables, respectively.

To account for potential confounding, propensity score matching was performed. Hernia repairs from both age groups were matched in a 1:1 ratio using a robust Greedy matching algorithm with a caliper width of 0.2 standard deviations of the logit of the propensity score.

The following matching variables were included in the propensity score model:

- BMI (underweight, normal weight, overweight, obese/morbid).
- ASA score (I, II, III–IV).
- Defect size (I < 1.5 cm, II 1.5–3 cm, III > 3 cm).
- EHS classification medial (yes/no).
- EHS classification lateral (yes/no).
- EHS classification femoral (yes/no).
- EHS classification scrotal (yes/no).
- Preoperative pain (yes/no/unknown).
- COPD (yes/no).
- Diabetes mellitus (yes/no).
- Aortic aneurysm (yes/no).
- Immunosuppression (yes/no).
- Corticoid therapy (yes/no).
- Smoking (yes/no).
- Coagulopathy (yes/no).
- Anticoagulant medication (yes/no).
- Antithrombotic medication (yes/no).

In addition, the following variables were defined as fixed matching variables with exact matching between pairs:

- Sex (male/female).
- Surgical approach (laparoscopic, open with mesh, open without mesh).
- Procedure type (elective, emergency with incarceration, emergency without incarceration).

Balance of matching variables before and after matching was assessed using standardized differences, with values < 10% (< 0.1) indicating adequate balance.

Additionally, we compared the study population with patients excluded due to missing follow-up data. Meaningful differences were observed only for the risk factor smoking, with a lower proportion of smokers in the analysis population.

The following outcome parameters were analysed in the matched-pair comparison (≥ 80 years vs. < 80 years): intraoperative complications, postoperative complications, general complications, complication-related reoperations, and one-year follow-up outcomes including pain at rest, pain on exertion, pain requiring treatment, recurrence, trocar hernia, secondary haemorrhage, seroma, and infection.

For categorical outcome variables, the exact McNemar test was used to assess systematic differences between paired patients. Results were expressed as adjusted odds ratios with 95% confidence intervals and presented in a forest plot.

AI-assisted language editing was used to improve clarity. The authors take full responsibility for the content.

## Results

### Study population

**Fig. 1:**
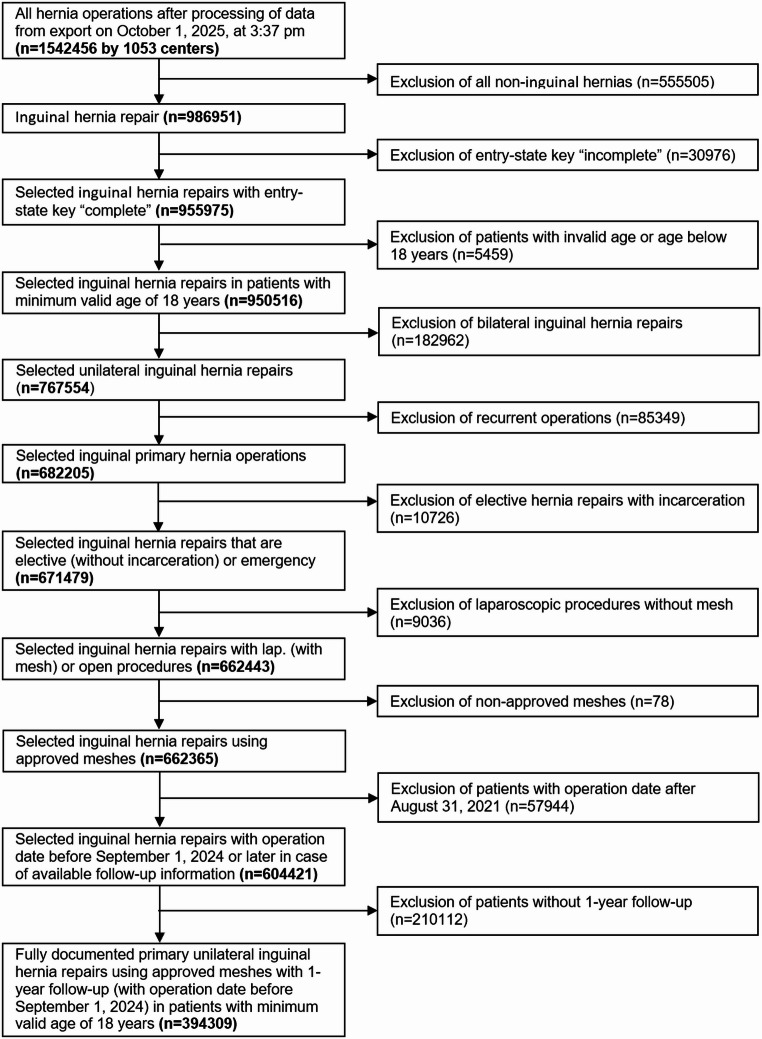
Patient enrolment flow chart for the study cohort

According to the study flow chart, a total of 394,309 patients were included in the statistical analyses (Fig. 1). Among these, 45,576 patients were aged ≥ 80 years (Table 1).

### Descriptive statistics and unadjusted analyses

In the overall cohort, differences in the distribution of patient and surgical characteristics between the age groups (≥ 80 years vs. < 80 years) were examined prior to matching. Supplementary Table 1 presents descriptive statistics and test results for the continuous variable operative duration, whereas Supplementary Table 2 summarises the corresponding analyses for categorical variables.

### Propensity score matching

Propensity score matching was performed for the 45,576 patients aged ≥ 80 years using the greedy matching algorithm with a caliper width of 0.2 standard deviations of the logit of the propensity score. Matching was conducted against the population of 348,733 patients aged < 80 years.

Successful matching to a control patient was achieved for 44,550 octogenarians (97.7%) (Table 2).

**Table 2:** Propensity score matching - number of matched pairs

Age ≥ 80 years	*N*_Matched_ / *N*_Original_	%
yes	44,550 / 45,576	97.7
no	44,550 / 348,733	12.8

### Balance of matching variables

**Fig. 2:**
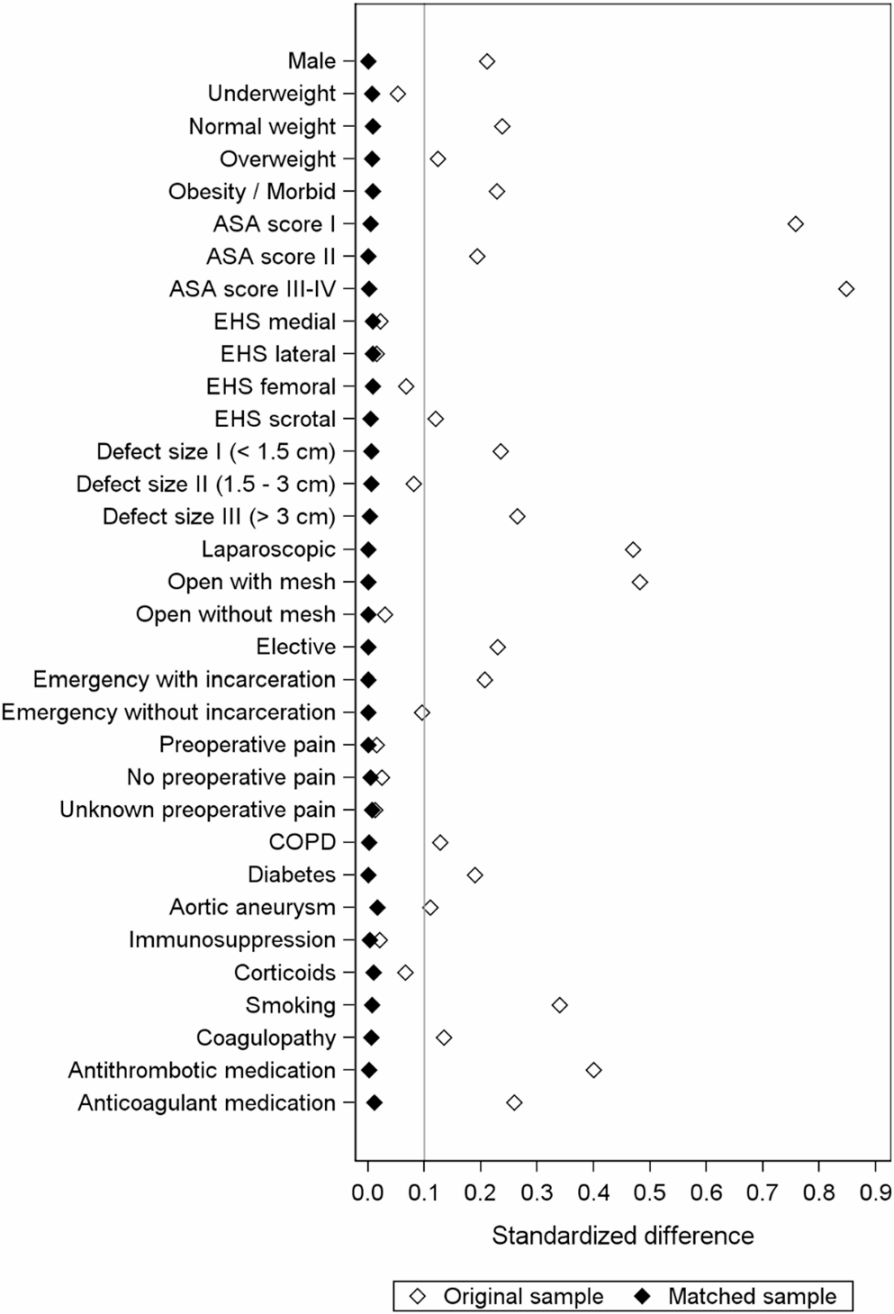
Scatter plot showing standardized differences of matching variables before and after propensity score matching (< 80 years vs. ≥ 80 years)

Standardized differences of the matching variables were assessed before (original cohort) and after matching (matched cohort). The standardized differences for the categorical matching variables are shown in Fig. 2; Table 3.

**Table 3:** Standardized differences before and after matching. Exact matching was enforced for sex, surgical technique, and type of procedure

		Age ≥ 80 years		Standardized difference	
Variable	Statistics	Yes	No	Matched sample	Original sample
Male*	N / %	36,551 / 82.0	36,551 / 82.0	0	0.211
Underweight	N / %	583 / 1.3	550 / 1.2	0.007	0.053
Normal weight	N / %	24,533 / 55.1	24,328 / 54.6	0.009	0.237
Overweight	N / %	17,057 / 38.3	17,211 / 38.6	0.007	0.123
Obesity / Morbid	N / %	2377 / 5.3	2461 / 5.5	0.008	0.228
ASA score I	N / %	1899 / 4.3	1861 / 4.2	0.004	0.757
ASA score II	N / %	20,301 / 45.6	20,304 / 45.6	< 0.001	0.193
ASA score III-IV	N / %	22,350 / 50.2	22,385 / 50.2	0.002	0.848
EHS medial	N / %	17,198 / 38.6	17,014 / 38.2	0.008	0.022
EHS lateral	N / %	32,262 / 72.4	32,428 / 72.8	0.008	0.016
EHS femoral	N / %	1884 / 4.2	1811 / 4.1	0.008	0.067
EHS scrotal	N / %	2601 / 5.8	2550 / 5.7	0.005	0.120
Defect size I (< 1.5 cm)	N / %	4120 / 9.2	4045 / 9.1	0.006	0.236
Defect size II (1.5–3 cm)	N / %	24,964 / 56.0	25,108 / 56.4	0.007	0.081
Defect size III (> 3 cm)	N / %	15,466 / 34.7	15,397 / 34.6	0.003	0.264
Laparoscopic*	N / %	20,014 / 44.9	20,014 / 44.9	0	0.470
Open with mesh*	N / %	23,858 / 53.6	23,858 / 53.6	0	0.481
Open without mesh*	N / %	678 / 1.5	678 / 1.5	0	0.031
Elective*	N / %	41,909 / 94.1	41,909 / 94.1	0	0.230
Emergency with incarceration*	N / %	2107 / 4.7	2107 / 4.7	0	0.207
Emergency without incarceration*	N / %	534 / 1.2	534 / 1.2	0	0.096
Preoperative pain	N / %	29,057 / 65.2	29,066 / 65.2	< 0.001	0.016
No preoperative pain	N / %	11,621 / 26.1	11,700 / 26.3	0.004	0.025
Unknown preoperative pain	N / %	3872 / 8.7	3784 / 8.5	0.007	0.012
COPD	N / %	3547 / 8.0	3517 / 7.9	0.002	0.128
Diabetes	N / %	4709 / 10.6	4711 / 10.6	< 0.001	0.190
Aortic aneurysm	N / %	717 / 1.6	625 / 1.4	0.017	0.111
Immunosuppression	N / %	485 / 1.1	468 / 1.1	0.004	0.021
Corticoids	N / %	714 / 1.6	657 / 1.5	0.010	0.067
Smoking	N / %	1238 / 2.8	1181 / 2.7	0.008	0.339
Coagulopathy	N / %	1502 / 3.4	1460 / 3.3	0.005	0.134
Antithrombotic medication	N / %	10,127 / 22.7	10,168 / 22.8	0.002	0.400
Anticoagulant medication	N / %	3015 / 6.8	2884 / 6.5	0.012	0.259

After matching, standardized differences for all matching variables were below 10%, indicating an adequate balance of the variables included in the propensity score model.

### Outcome analysis

Table 4 summarises the results of the matched-pair comparisons between patients aged ≥ 80 years and those aged < 80 years across the predefined outcome parameters.

**Table 4:** Results of the matched-pair comparison for systematic differences between age groups (≥ 80 years vs. < 80 years) (*n* = 44,550 paired operations)

	Concordantcases *N* (%)		Discordantcases *N* (%)			OR for matched samples		
Outcome Variables	Event in both patients	No event in both patients	Event in 'Age ≥ 80 years' only	Event in 'Age < 80 years' only	*p*-Value	OR	Lower limit	Upper limit
Intraoperative complications	4 (<0.1%)	43724 (98.1%)	400 (0.9%)	422 (0.9%)	0.464	0.948	0.825	1.089
General complications	22 (< 0.1%)	42,955 (96.4%)	975 (2.2%)	598 (1.3%)	< 0.001	1.630	1.471	1.808
Postoperative complications	108 (0.2%)	40,877 (91.8%)	1962 (4.4%)	1603 (3.6%)	< 0.001	1.224	1.145	1.308
Complication-related reoperations	14 (< 0.1%)	43,229 (97.0%)	737 (1.7%)	570 (1.3%)	< 0.001	1.293	1.158	1.445
Recurrence on 1-year follow-up	5 (< 0.1%)	43,627 (97.9%)	428 (1.0%)	490 (1.1%)	0.044	0.873	0.765	0.996
Pain on exertion on 1-year follow-up	197 (0.4%)	38,739 (87.0%)	1962 (4.4%)	3652 (8.2%)	< 0.001	0.537	0.508	0.568
Pain at rest on 1-year follow-up	66 (0.1%)	41,159 (92.4%)	1241 (2.8%)	2084 (4.7%)	< 0.001	0.595	0.555	0.639
Pain requiring treatment on 1-year follow-up	24 (< 0.1%)	42,541 (95.5%)	772 (1.7%)	1213 (2.7%)	< 0.001	0.636	0.581	0.697
Trocar hernia on 1-year follow-up	0 (0%)	44,406 (99.7%)	67 (0.2%)	77 (0.2%)	0.453	0.870	0.618	1.223
Secondary hemorrhage on 1-year follow-up	18 (< 0.1%)	42,801 (96.1%)	913 (2.0%)	818 (1.8%)	0.024	1.116	1.015	1.228
Seroma on 1-year follow-up	26 (< 0.1%)	42,530 (95.5%)	1024 (2.3%)	970 (2.2%)	0.235	1.056	0.966	1.154
Infection on 1-year follow-up	7 (< 0.1%)	43,517 (97.7%)	560 (1.3%)	466 (1.0%)	0.004	1.202	1.061	1.362

A systematic difference between the age groups was observed for general complications, postoperative complications, complication-related reoperations, as well as for recurrence rates and pain outcomes at one-year follow-up. In addition, differences were identified for secondary postoperative bleeding and infections.

For general complications there is a significant deviation to patients aged ≥ 80 years in discordant cases (2.2% vs. 1.3%; *p* < 0.001, 22 (< 0.1%) concordant cases). This means that in total, 975 cases (2.2%) of general complications were observed in patients aged ≥ 80 years that did not occur in the matched patients aged < 80 years. Conversely, 598 cases (1.3%) of general complications occurred in patients aged < 80 years but not in their matched counterparts aged ≥ 80 years. Twenty-two concordant cases were observed, indicating matched pairs in which both patients experienced a general complication.

Similarly, significant deviations to the disadvantage of patients aged ≥ 80 years were observed for postoperative complications (4.4% vs. 3.6%; *p* < 0.001, 108 (0.2%) concordant cases), complication-related reoperations (1.7% vs. 1.3%; *p* < 0.001, 14 (< 0.1%) concordant cases), postoperative bleeding (2.0% vs. 1.8%; *p* = 0.024, 18 (< 0.1%) concordant cases), and infections (1.3% vs. 1.0%; *p* = 0.004, 7 (< 0.1%) concordant cases) at one-year follow-up.

In contrast, there is a significant deviation in favour of patients aged ≥ 80 years for recurrences (1.0% vs. 1.1%; *p* = 0.044, 5 (< 0.1%) concordant cases), pain on exertion (4.4% vs. 8.2%; *p* < 0.001, 197 (0.4%) concordant cases), pain at rest (2.8% vs. 4.7%; *p* < 0.001, 66 (0.1%) concordant cases) and pain requiring treatment (1.7% vs. 2.7%; *p* < 0.001, 24 (< 0.1%) concordant cases).

No significant systematic deviation between the matched age groups were found for intraoperative complications or for the occurrence of trocar hernia and seroma during the one-year follow-up.

**Fig. 3:**
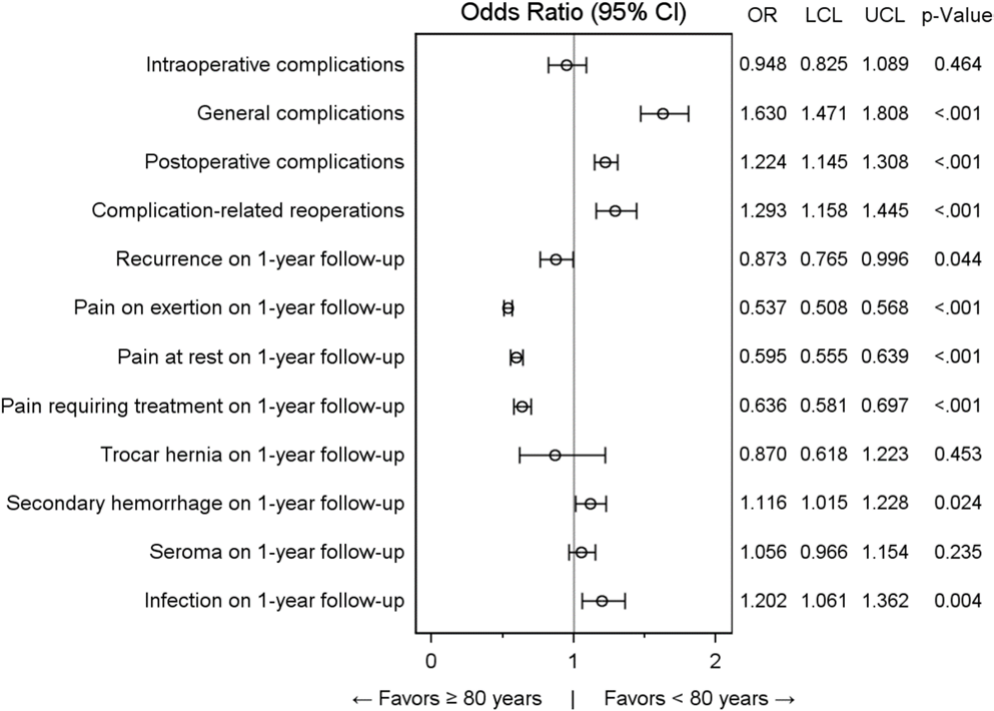
Forest plot showing adjusted odds ratios with 95% confidence intervals for all outcome parameters comparing patients aged ≥ 80 years with those aged < 80 years

For improved visualization, the results are presented in Fig. 3 as odds ratios for matched pairs with corresponding 95% confidence intervals. Confidence intervals entirely below 1 indicate a significant disadvantage for patients aged < 80 years, whereas intervals entirely above 1 indicate a disadvantage for patients aged ≥ 80 years.

## Discussion

This study compared patients aged ≥ 80 years with those aged < 80 years using data from the Herniamed Registry, focusing on hernia characteristics, anaesthesia techniques, surgical approaches, and postoperative outcomes.

Increased complication rates in elderly patients have previously been reported for other hernia types. For example, a study based on the Herniamed Registry demonstrated higher complication rates for incisional hernia repair in elderly patients [23].

Current international guidelines do not provide separate recommendations for elderly patients but instead emphasize individualized treatment strategies based on hernia characteristics and patient comorbidities [24, 25]. Our registry analysis confirms a higher proportion of emergency procedures among patients aged ≥ 80 years. In our view, this likely contributes to the increased rates of perioperative complications observed in this population. Several studies have reported a higher risk of emergency surgery with increasing age, which is associated with substantially increased morbidity and mortality [11, 12, 26, 27]. Consequently, prompt elective repair after diagnosis of an inguinal hernia is recommended even in octogenarians in order to prevent emergency situations [13, 28].

Considering the potential risk of postoperative cognitive dysfunction, inguinal hernia repair in elderly patients can also be performed under local anaesthesia [29]. Local anaesthesia offers advantages such as faster recovery and facilitates outpatient management, making it particularly suitable for many octogenarians [30].

With regard to the surgical approach, numerous studies suggest that laparo-endoscopic and open techniques provide comparable overall outcomes, and both approaches appear feasible in octogenarians [31]. Nevertheless, the use of laparo-endoscopic procedures is clearly lower in patients aged ≥ 80 years. This may be explained by several factors, including the higher prevalence of large or complex hernias, a greater burden of comorbidities, and a higher proportion of emergency procedures in this age group. Several authors therefore recommend an individualized approach depending on patient characteristics and hernia morphology. For example, a higher ASA classification or the presence of scrotal hernias may favour open repair, whereas bilateral hernias are often considered more suitable for laparo-endoscopic techniques [32].

In general, laparo-endoscopic procedures have been associated with higher rates of postoperative seroma and urinary retention compared with open techniques [33]. However, laparo-endoscopic repair offers specific advantages, including the possibility of detecting contralateral hernias intraoperatively [34]. Several studies have demonstrated that laparo-endoscopic surgery in octogenarians does not lead to increased complication rates [34, 35] or higher morbidity and mortality [36]. Some authors therefore conclude that, compared with open repair, laparo-endoscopic techniques may offer advantages with regard to postoperative pain and recovery of daily activities and should also be considered in elderly patients [37]. A recently published meta-analysis reported no significant differences in postoperative complications between open and endoscopic repair in octogenarians; however, the incidence of general complications was significantly higher in the laparoscopic group than in the open group [38].

Most inguinal hernia repairs in octogenarians can also be performed in an outpatient setting. Previous studies have reported no relevant differences in planned discharge rates [39] or postoperative complication rates following laparo-endoscopic repair [40]. Nevertheless, some studies suggest that readmission rates after outpatient laparo-endoscopic hernia repair may be higher in octogenarians [41].

### Limitations

Several limitations of the present study should be acknowledged. First, statistically significant differences do not necessarily imply clinical relevance. Given the large sample size, even small differences may achieve statistical significance. Therefore, the clinical relevance of these findings should be interpreted with caution, particularly as the analyses were conducted in an exploratory manner at the full significance level.

**Fig. 4:**
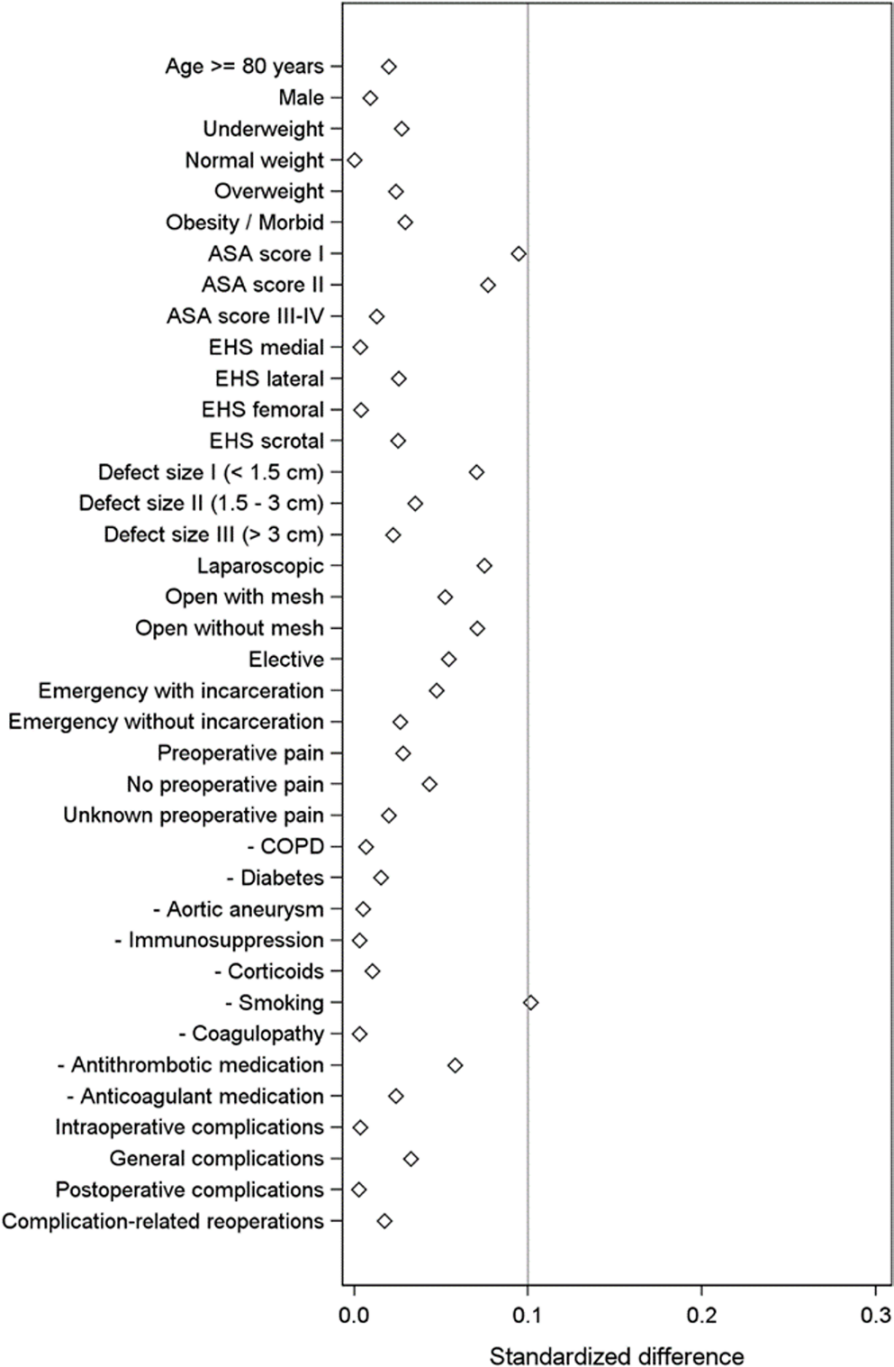
Scatter plot showing standardized differences between patients with and without one-year follow-up

Second, missing one-year follow-up data for a proportion of patients represents another limitation. However, calculation of standardized differences between patients with and without follow-up did not indicate relevant selection bias (Fig. 4).

In addition, the groups differed substantially in size before matching. Although propensity score matching was applied to minimize confounding, residual confounding cannot be completely excluded in registry-based analyses. Patient-reported outcomes such as pain may also be subject to reporting bias or influenced by differences in activity levels between age groups. Moreover, the conclusions apply only to the matched population and may not be generalizable to patients with extreme risk profiles who could not be matched. Finally, although the one-year follow-up provides important insights, longer-term outcomes beyond one year were not evaluated in the present analysis.

The assessment of pain sensitivity in older individuals is inherently limited. The lower postoperative pain rates observed in octogenarians may be attributable to several factors, including diminished pain perception with advancing age and reduced levels of physical activity. However, pain assessment is intrinsically subjective and may vary both between patients and among observers. Furthermore, the potential influence of reporting bias and survivorship bias must be considered.

Another potential limitation relates to variations in surgical experience and case volumes across participating hernia centres. Nevertheless, given the large number of patients included in the registry, the statistical impact of such differences is likely to be limited.

Overall, these real-world data suggest that although octogenarians have an increased perioperative risk, elective inguinal hernia repair can be safely integrated into routine clinical practice when individualized perioperative management is applied. Chronological age alone should therefore not be considered a contraindication to surgical treatment.

## Conclusion

In this large registry-based analysis using propensity score matching, patients aged ≥ 80 years undergoing inguinal hernia repair exhibited higher rates of general and postoperative complications, reoperations, bleeding, and infections compared with younger patients, even after adjustment for patient-, hernia-, and procedure-related factors. They more frequently presented with larger defects and emergency indications and were more often managed with open mesh repair under regional or local anaesthesia, reflecting greater surgical complexity and perioperative vulnerability.

Despite these risks, elective repair in octogenarians was safe and effective when individualized perioperative management was applied. Chronological age alone should not preclude surgical treatment. The higher rate of emergency procedures underscores the importance of timely elective intervention, as emergency surgery carries substantially increased risk. When feasible, repair should be performed at an earlier stage to avoid progression to higher-risk scenarios.

Notably, one-year outcomes—including recurrence and patient-reported pain—were more favorable in octogenarians than in younger patients. Overall, these findings support the integration of elective inguinal hernia repair into routine care for elderly patients with appropriate risk stratification.

Future studies should incorporate bilateral hernias and further evaluate age-related factors such as frailty, functional reserve, and cognitive status.

## Supplementary information

Below is the link to the electronic supplementary material.

Supplementary File 1 (DOCX 13.5 KB)

Supplementary File 2 (DOCX 22.4 KB)
