# Supplementary material for: Outcomes of inguinal hernia repair in octogenarians: A propensity score–matched analysis of the Herniamed Registry
Source: Hernia. 2026 May 12;30(1):203. doi: 10.1007/s10029-026-03689-5 (PMC13167829; doi:10.1007/s10029-026-03689-5)
Supplement: Supplementary file 1 — (DOCX 13.5 KB) [file 10029_2026_3689_MOESM1_ESM.docx]

|  | | **Age ≥ 80 years** | |  |
| --- | --- | --- | --- | --- |
|  | | **Yes** | **No** |  |
|  | | | | **p** |
| Duration of operation [min]* | N / Mean [Range of dispersion] | 45,079 / 52.6 [35.3; 78.4] | 343,765 / 49.8 [33.2; 74.5] | <.001 |

Suppl. Tab. 1 Distribution ranges and results of the unadjusted homogeneity analysis between the age groups (≥80 years vs <80 years) for operative duration before propensity score matching.

*Logarithmic transformation: presentation of back-transformed mean values and ranges (mean ± SD).
