# Supplementary material for: Outcomes of inguinal hernia repair in octogenarians: A propensity score–matched analysis of the Herniamed Registry
Source: Hernia. 2026 May 12;30(1):203. doi: 10.1007/s10029-026-03689-5 (PMC13167829; doi:10.1007/s10029-026-03689-5)
Supplement: Supplementary file 2 — (DOCX 22.4 KB) [file 10029_2026_3689_MOESM2_ESM.docx]

|  | | **Age ≥ 80 years** | | | |  |
| --- | --- | --- | --- | --- | --- | --- |
|  | | **Yes** | | **No** | |  |
|  | | **n** | **%** | **n** | **%** | **p** |
|  | | | | | | |
| Sex | Male | 36787 | 80.7 | 307991 | 88.3 | <.001 |
|  | Female | 8789 | 19.3 | 40742 | 11.7 |  |
|  | | | | | | |
| Type of access | Laparoscopic | 20265 | 44.5 | 234256 | 67.2 | <.001 |
|  | Open with mesh | 24555 | 53.9 | 107253 | 30.8 |  |
|  | Open without mesh | 756 | 1.7 | 7224 | 2.1 |  |
|  | | | | | | |
| Type of procedure | Elective | 42486 | 93.2 | 341459 | 97.9 | <.001 |
|  | Emergency with incarceration | 2415 | 5.3 | 5429 | 1.6 |  |
|  | Emergency without incarceration | 675 | 1.5 | 1845 | 0.5 |  |
|  | | | | | | |
| BMI | Underweight | 625 | 1.4 | 2853 | 0.8 | <.001 |
|  | Normal weight | 25175 | 55.4 | 151807 | 43.7 |  |
|  | Overweight | 17226 | 37.9 | 152923 | 44.0 |  |
|  | Obesity / Morbid | 2378 | 5.2 | 40023 | 11.5 |  |
|  | | | | | | |
| ASA | I | 1905 | 4.2 | 108832 | 31.2 | <.001 |
|  | II | 20408 | 44.8 | 189667 | 54.4 |  |
|  | III/IV | 23263 | 51.0 | 50234 | 14.4 |  |
|  | | | | | | |
| Defect size (inguinal) | I (< 1.5 cm) | 4192 | 9.2 | 59641 | 17.1 | <.001 |
|  | II (1.5 - 3 cm) | 25500 | 56.0 | 208986 | 59.9 |  |
|  | III (> 3 cm) | 15884 | 34.9 | 80106 | 23.0 |  |
|  | | | | | | |
| EHS medial | yes | 17588 | 38.6 | 130871 | 37.5 | <.001 |
|  | no | 27988 | 61.4 | 217862 | 62.5 |  |
|  | | | | | | |
| EHS lateral | yes | 32896 | 72.2 | 254170 | 72.9 | 0.001 |
|  | no | 12680 | 27.8 | 94563 | 27.1 |  |
|  | | | | | | |
| EHS femoral | yes | 2026 | 4.4 | 11037 | 3.2 | <.001 |
|  | no | 43550 | 95.6 | 337696 | 96.8 |  |
|  | | | | | | |
| EHS scrotal | yes | 2639 | 5.8 | 11471 | 3.3 | <.001 |
|  | no | 42937 | 94.2 | 337262 | 96.7 |  |
|  | | | | | | |
| Preoperative pain | no | 11785 | 25.9 | 93958 | 26.9 | <.001 |
|  | yes | 29841 | 65.5 | 225738 | 64.7 |  |
|  | unknown | 3950 | 8.7 | 29036 | 8.3 |  |
|  | | | | | | |
| Anesthesia | Local | 1147 | 2.5 | 2346 | 0.7 | <.001 |
|  | Spinal | 3078 | 6.8 | 8294 | 2.4 |  |
|  | General | 41351 | 90.7 | 338093 | 96.9 |  |
|  | | | | | | |
| Fixation | No Mesh Fixation | 16995 | 38.1 | 176072 | 51.8 | <.001 |
|  | Tacks + Suture + Glue | 5 | <0.1 | 23 | <0.1 |  |
|  | Tacks + Suture | 72 | 0.2 | 620 | 0.2 |  |
|  | Tacks + Glue | 87 | 0.2 | 705 | 0.2 |  |
|  | Suture + Glue | 524 | 1.2 | 2571 | 0.8 |  |
|  | Tacks | 2659 | 6.0 | 26776 | 7.9 |  |
|  | Suture | 20858 | 46.8 | 94902 | 27.9 |  |
|  | Glue | 3388 | 7.6 | 38405 | 11.3 |  |
|  | | | | | | |
| Risk factors - total | yes | 21360 | 46.9 | 99788 | 28.6 | <.001 |
|  | no | 24216 | 53.1 | 248945 | 71.4 |  |
|  | | | | | | |
| - COPD | yes | 3641 | 8.0 | 16923 | 4.9 | <.001 |
|  | no | 41935 | 92.0 | 331810 | 95.1 |  |
|  | | | | | | |
| - Diabetes | yes | 4819 | 10.6 | 19012 | 5.5 | <.001 |
|  | no | 40757 | 89.4 | 329721 | 94.5 |  |
|  | | | | | | |
| - Aortic aneurysm | yes | 735 | 1.6 | 1688 | 0.5 | <.001 |
|  | no | 44841 | 98.4 | 347045 | 99.5 |  |
|  | | | | | | |
| - Immunosuppression | yes | 493 | 1.1 | 3056 | 0.9 | <.001 |
|  | no | 45083 | 98.9 | 345677 | 99.1 |  |
|  | | | | | | |
| - Corticoids | yes | 741 | 1.6 | 3076 | 0.9 | <.001 |
|  | no | 44835 | 98.4 | 345657 | 99.1 |  |
|  | | | | | | |
| - Smoking | yes | 1242 | 2.7 | 39243 | 11.3 | <.001 |
|  | no | 44334 | 97.3 | 309490 | 88.7 |  |
|  | | | | | | |
| - Coagulopathy | yes | 1555 | 3.4 | 4766 | 1.4 | <.001 |
|  | no | 44021 | 96.6 | 343967 | 98.6 |  |
|  | | | | | | |
| - Antithrombotic medication | yes | 10445 | 22.9 | 30029 | 8.6 | <.001 |
|  | no | 35131 | 77.1 | 318704 | 91.4 |  |
|  | | | | | | |
| - Anticoagulant medication | yes | 3171 | 7.0 | 6009 | 1.7 | <.001 |
|  | no | 42405 | 93.0 | 342724 | 98.3 |  |

Suppl. Tab. 2 Descriptive statistics and results of the unadjusted homogeneity tests between the age groups (≥80 years vs <80 years) for categorical variables before propensity score matching.
